# New York Letter

**Published:** 1896-01

**Authors:** Ogden C. Ludlow

**Affiliations:** 2309 Seventh Avenue; New York


### *CORRESPONDENCE.*

---

### NEW YORK LETTER.

New York, December 15, 1895.

I trust I may be pardoned for referring again to the antitoxin treatment of diphtheria. It is a subject that will not "down," at least here, and the medical societies vie with each other in arranging more or less elaborate "antitoxin tournaments." At one of the most recent of these the conflict raged fiercely and long, and the degree of interest that was aroused may be guessed when I say that the scientific session lasted until midnight, and that, too, in the face of the fact that, in the adjoining room, weary caterers were waiting to serve a collation. Comment is unnecessary.

Among the many papers on this subject was one entitled, "The Present Status of the Diphtheria Question," read by Dr. Louis Fischer. He had observed over five hundred cases treated with the diphtheria antitoxin, and he was now prepared to reiterate his former statements regarding the great value of this method of treatment. He had not had a single death in his last sixty-four cases, notwithstanding that many of these were very malignant, and in forty of them intubation had been required. In about twenty per cent. albuminuria had been observed, and in some cases there had also been hematuria. It had been noted that in some instances the quantity of albumen in the urine had suddenly increased immediately after a second injection of antitoxin, and from this he had at first been led to think that the albuminuria was due to certain impurities in the antitoxin. He now felt convinced, however, that the albuminuria was in reality a part of the diphtheria in these severe cases, and that, in all probability, it would have been present had the antitoxin not been administered at all. In using the antitoxin treatment, it had been his rule to administer a second injection of the serum at the end of twenty-four hours, if no improvement had followed from the first dose. He had noted no constitutional symptoms following the injection of the antitoxin, with the exception of a slight rise of temperature, occurring in the first few hours. Contrary to the opinion quite generally expressed by other observers, Dr. Fischer holds that the antitoxin treatment is decidedly beneficial in the cases of mixed infection. If this had been the experience of many physicians, I am sure that antitoxin would have much stronger claims for recognition, but, unfortunately, this experience is decidedly unique. Even the most ardent advocates of the new treatment have been compelled to admit that diphtheria antitoxin is only potent in controlling the toxemia of pure diphtheria, and that it is powerless, or nearly so, in the very class of cases in which we would wish most to invoke its aid, *i. e.*, the mixed infections. If it were necessary to emphasize this point still further, reference might be made to the recent remarks of Dr. J. Lewis Smith before the Academy of Medicine, in which he stated that he had found in the extensive diphtheria service of the foundling asylum that antitoxin had acted well in the moderately severe cases of pure diphtheria, but had been most disappointing in the very severe cases; in other words, those due to mixed infection. He very properly added that if some method of successfully treating this mixed infection class were devised, there would be little cause for anxiety about the cases of pure Klebs-Lœffler bacillus diphtheria.

Up to a short time ago there was but one voice here in our meetings raised against the treatment of diphtheria with antitoxin, but gradually the opponents of this method have increased in numbers and strength, and we no longer listen to "eulogies" under the name of "discussions." For example, since Dr. William Vissman has shown, by a careful series of experiments on animals, that injections of antitoxin in health cause quite uniformly pathological changes in the kidneys; and since Dr. H. D. Chapin has shown by his experiments that horse-serum itself is by no means inert when introduced into the circulation of animals, there have been a number of physicians who have hesitated to treat their cases of diphtheria with antitoxin. In the discussion of Dr. Fischer's paper, Dr. William Henry Porter said that he objected to the use of antitoxin because, in the effort to eliminate it from the system, the kidneys were apparently overtaxed and injured. He also felt convinced that the much-quoted statistics did not truthfully represent the position of diphtheria antitoxin as a therapeutic agent. Although there had been no epidemics of diphtheria in this city during the past year, the number of reported cases of diphtheria was much larger than in those years in which there had been epidemics of this disease. This was attributable to the new regulations of the Board of Health regarding establishing the diagnosis of diphtheria by bacteriological examination. If many cases in which the Klebs bacilli were found, but which were not clinically examples of diphtheria, were included in the statistics, it was evident that there would be a marked reduction in the mortality from this disease. So far as he knew, these cases had been included in the statistical studies that had been made concerning diphtheria antitoxin. There were two other sources of grave error which had been introduced, viz.: the exclusion from consideration of the many and necessarily fatal cases of diphtheria with mixed infection and the comparison of the present death rate from diphtheria with that of a number of years ago, when our treatment was not nearly so successful as it had been just prior to the introduction of antitoxin. In his opinion, diphtheria antitoxin was doomed to meet the same fate as tuberculin. Dr. William Vissman, in continuing this discussion, referred to the experiments described in the work on physiology by Landois and Stirling, in which it was shown that the transfusion of heterogeneous serum and blood produced very nearly the same changes in the internal viscera as were caused by a severe attack of diphtheria. He also called attention to his own experiments on animals, which indicated that the subcutaneous introduction of diphtheria antitoxin produced analagous lesions.

Dr. J. C. Smith, of the laboratory of the Post-Graduate school, took occasion at this time to say that he believed that the whole matter of the bacteriological diagnosis of diphtheria was entirely premature, and that it was his opinion that a year from now hardly any one would be using diphtheria antitoxin.

This question of the value of bacteriological examinations in establishing the diagnosis of diphtheria is one which is puzzling many physicians, at least in New York city. There has been abundant opportunity, one would think, to demonstrate the value of such examinations as made by the Board of Health, yet it is a fact that many in the rank and file of the medical profession, although not outspoken on this point, are coming to feel that it is indeed "a broken reed" to lean upon. Whatever may be said about the theoretical advantages of having the clinical diagnosis strengthened or disproved by a bacteriological examination, and however interesting it may be as a matter of scientific record under certain circumstances, there can be no question about its practical absurdities, as exemplified in its application by our Board of Health. For example, the writer recalls a case presenting all the clinical evidences of a genuine diphtheria of the nose and throat, in which he took a culture from the throat and submitted it to the Board of Health for a bacteriological examination. The report from the laboratory stated that it was true diphtheria. It ran the usual course of a rather severe diphtheria, and during this time the Inspector of the Health Department also had a culture examined, and reported it to be a genuine case of this disease. In order to test the practical value of these examinations, and of the regulations of the Board founded upon them, I took great pains to thoroughly swab the upper part of the pharynx, and send a culture from it to the Health Department just as the membrane had cleared off the throat, but while huge plaques of membrane were being frequently discharged from the nose. To my astonishment I received a report the next day to the effect that as this last culture no longer showed the presence of the diphtheria bacilli, the place was ready for disinfection, and they would attend to this without delay. Of course, I explained to the family the absurdity of such a procedure, and stated that I would attend to the final disinfection, even though the Health Department had already fumigated the rooms.

Again, it is a violation of the sanitary Code for a physician to send a supposed case of diphtheria to the Willard-Parker Hospital in a street car or other public conveyance, but if he meets with a case in his practice, and desires to have it transferred to the Hospital in their ambulance, he will find that he cannot do so until one or more representatives of the Health Department has visited the patient, and a culture has been taken and examined; and not even then, if it happens that the Klebs-Lœffler bacilli are not in evidence. It matters not whether there is a membranous exudate in the throat, whether there is necrosis, whether there is grave constitutional disturbance—in short, all the clinical evidences of diphtheria—that individual must remain where he or she is for twenty-four or forty-eight hours until the Board of Health has decided whether or not it is a case of diphtheria. All this time the sick person may be exposing a whole family or more to infection, but that is of no consequence in comparison with establishing a bacteriological diagnosis.

Another crying shame in connection with this same question, is the practice of the inspectors coming in and personally taking cultures from the throat. This has been done even where the attending physician has already sent one to the Board for examination, and in cases in which he has requested that the patient be not disturbed by the inspectors. It is no trifling matter for a petted, delicate, nervous child, already prostrated with diphtheria, to be suddenly confronted in its own home by a stranger, who orders it to open its mouth while he proceeds to swab the throat in no very gentle way. I have known this high-handed procedure to throw a child into such a condition of collapse that hypodermic injections of brandy and other vigorous measures were necessary to save the little one's life.

Many other equally striking examples might easily be collected to show the follies and wrongs connected with this practice. I do not wish to be understood as opposing legitimate efforts to secure scientific accuracy, but I do earnestly desire to protest most vehemently against the commission of such wrongs in the name of science and humanity. This practice has already led to one unfortunate result—that of bringing our Health Department into contempt in the eyes of many, both in and out of the medical profession.

OGDEN C. LUDLOW, M.D.

*2309 Seventh Avenue.*

